# Policy Action Within Urban African Food Systems to Promote Healthy Food Consumption: A Realist Synthesis in Ghana and Kenya

**DOI:** 10.34172/ijhpm.2020.255

**Published:** 2021-02-09

**Authors:** Andrew Booth, Amy Barnes, Amos Laar, Robert Akparibo, Fiona Graham, Kristin Bash, Gershim Asiki, Michelle Holdsworth

**Affiliations:** ^1^School of Health and Related Research (ScHARR), University of Sheffield, Sheffield, UK.; ^2^School of Public Health, University of Ghana, Accra, Ghana.; ^3^Institute for Health and Society, Newcastle University, Newcastle upon Tyne, UK.; ^4^African Population and Health Research Center (APHRC), Nairobi, Kenya.; ^5^French National Research Institute for Sustainable Development (IRD), NUTRIPASS Unit, IRD-Univ Montpellier, Montpellier, France.

**Keywords:** Africa, Food Environments, Food Policy, Food Consumption, Realist Review

## Abstract

**Background:** Obesity and nutrition-related non-communicable diseases (NR-NCDs) are increasing throughout Africa, driven by urbanisation and changing food environments. Policy action has been limited - and influenced by high income countries. Socio-economic/political environments of African food systems must be considered in order to understand what policy might work to prevent NR-NCDs, for whom, and under what circumstances.

**Methods:** A realist synthesis of five policy areas to support healthier food consumption in urban Africa: regulating trade/foreign investment; regulating health/nutrition claims/labels; setting composition standards for processed foods; restricting unhealthy food marketing; and school food policy. We drew upon Ghana and Kenya to contextualise the evidence base. Programme theories were generated by stakeholders in Ghana/Kenya. A two-stage search interrogated MEDLINE, Web of Science and Scopus. Programme theories were tested and refined to produce a synthesised model.

**Results:** The five policies operate through complex, inter-connected pathways moderated by global-, national- and local contexts. Consumers and the food environment interact to enable/disable food accessibility, affordability and availability. Consumer relationships with each other and retailers are important contextual influences, along with political/ economic interests, stakeholder alliances and globalized trade. Coherent laws/regulatory frameworks and government capacities are fundamental across all policies. The increasing importance of convenience is shaped by demographic and sociocultural drivers. Awareness of healthy diets mediates food consumption through comprehension, education, literacy and beliefs. Contextualised data (especially food composition data) and inter-sectoral collaboration are critical to policy implementation.

**Conclusion:** Evidence indicates that coherent action across the five policy areas could positively influence the healthiness of food environments and consumption in urban Africa. However, drivers of (un)healthy food environments and consumption reflect the complex interplay of socio-economic and political drivers acting at diverse geographical levels. Stakeholders at local, national, and global levels have important, yet differing, roles to play in ensuring healthy food environments and consumption in urban Africa.

## Background

Many African countries are experiencing a nutrition transition with shifting dietary patterns, related to rapid urbanisation and changing food systems.^1^ Consequently, obesity and nutrition-related non-communicable diseases (NR-NCDs) are rapidly increasing; becoming an important public health challenge.^2^ Ghana and Kenya exemplify trends in rapid urbanisation and shifting dietary patterns.^3-5^ Rising overweight and obesity sit alongside persistent and significant burdens of under-nutrition and multiple micronutrient deficiencies in many African countries^3^; and thus this “multiple burden of malnutrition”^3,6^ presents a considerable challenge within African food systems, particularly within urban food environments.^7^ The food environment merits particular attention as the interface that mediates people’s food acquisition and consumption within the wider food system, that is influenced by policy and governance.^8^ Preventive policy action to address obesity and NR-NCDs within African urban food systems has been limited. High-level political commitments and strategies have been made on hunger and food security (eg, the 2014 Malabo Declaration,^9^ the Africa Region Nutrition Strategy 2015-2025,^10^ and Agenda 2063.^11^ Yet integrated efforts by African governments to address NCDs are scant, poorly coordinated and/or incompletely implemented. Research in Ghana and Kenya has, for example, identified gaps in policy implementation to promote healthy food environments, particularly in relation to food advertising (except marketing breastmilk), food trade, food retail, food prices, food provision (except schools).^12,13^ At the same time, evidence to inform action within food systems tends to be drawn from potentially less-relevant higher-income settings, partly due to data poverty in many African contexts. What works to enable healthy food consumption in one context need not indicate what could work in another. This lack of contextually-sensitive evidence on what could work in urban Africa undermines the transformative changes that will be required to create healthier food environments and healthier consumption.

Transforming food systems to address the rising challenge of NR-NCDs, particularly in urban Africa, needs to be driven by well-governed policy that considers whether decisions would support or undermine changes elsewhere in the food system. Policy-makers need evidence on what might work, and for whom, in specific urban contexts, if it is to support their decision-making and to inform the political trade-offs that are inevitably needed to secure transformational food system change.^14,15^ In practice, this means that evidence to inform strategies to support healthier food consumption, particularly in urban Africa, needs to account for the diverse socio-economic and political environments that drive unhealthy food consumption.

We carried out a realist synthesis against this backdrop, to contextualise what policy might work to prevent NR-NCDs in urban Africa, for whom, and under what circumstances. Given that food systems are diverse culturally, socio-economically and politically, we drew upon two African countries: – Ghana and Kenya – to contextualise the evidence base. We selected these two countries because they typify dietary and epidemiological transition seen in urban Africa,^3,4,16,17^ and their governments have recognised NR-NCDs as a pressing public health concern through the development of national policies to prevent NR-NCDs.^18,19^ We focused on evidence relating to five ‘good preventative policy’ actions from those identified internationally^20,21^ and locally^12,13^ to improve the healthiness of food systems: regulation of trade in goods/services and foreign direct investment (FDI); regulation of food health/nutrition claims; setting food composition standards/targets for processed foods; restricting the promotion of unhealthy food; and clear/consistent healthy food promotion policy in schools.

## Methods

Realist synthesis is an “approach to reviewing research evidence on complex social interventions, which provides an explanatory analysis of how and why they work (or do not work) in particular contexts or settings” (‘programme theory’; PT).^22^ By extension, realist analysis offers insights on whether similar action might work in other contexts.^23^ Policy actions are complex interventions that operate across multiple levels of interconnected food systems. A realist synthesis approach thus aligned with our research objectives in exploring ‘context’ at diverse levels; for example, community environments, cultures, and organisational structures, as well as wider socio-economic and political conditions.^24^

A realist synthesis starts by generating programme theories for specific policy actions from a scoping search of relevant literature. Empirical evidence is then examined to ‘test’ how theory relates to practice.^22^ Our realist synthesis followed a ‘realist template,’^25^ involving 4 main steps (see Table 1) and is reported according to RAMESES guidance.^26^ Our initial overarching question was: ‘What policies could support healthier food consumption in urban Africa, using Ghana and Kenya as examples to contextualise what might work, for whom, and under what circumstances?’ Potentially, the policy action landscape is broad, we therefore needed to identify focal areas of importance to Ghana and Kenya.

**Table 1:** Stages of the Realist Review

**Stage**	**Task**
1. Define the scope of the review	Identify the question Clarify the purpose(s) of the review Find and articulate the programme theories
2. Search for and appraise the evidence	Search for the evidence Appraise the evidence
3. Extract and synthesise findings	Extract the results Synthesise findings
4. Draw conclusions and make recommendations	

Adapted from Pawson.^25^

As a starting point we focused on the seven policy domains (eg, food labelling, promotion, composition, provision, retail, prices, trade and investment) and associated good practice indicators identified in the Healthy Food Environment Policy Index (Food-EPI): a tool developed by INFORMAS (International Network for Food and Obesity/Non-communicable Diseases Research, Monitoring and Action Support) for monitoring government action to improve food environments.^16^ The research team systematically gathered evidence on government action in relation to each policy domain/good practice indicator in Ghana and Kenya, as part of wider work to apply the Food-EPI in both countries.^12,13^ As part of this process, the Ghanaian and Kenyan research teams also consulted informally with key stakeholders in each country about the implementation of different actions locally, including with government officials and representatives of non-government organisations who had relevant expertise (eg, in nutrition, trade, academia, advocacy). Each country team used insights from these stakeholder discussions, alongside evidence identified in the Food-EPI policy review, to independently short list seven policies (one from each Food-EPI policy domain) for possible investigation in the realist review. The country teams then compared their short lists, identifying five focal policies for the realist review where there was complete overlap:

- Regulate trade in goods/services and FDI (PT1)
- Regulate food health/nutrition claims (PT2)
- Set food composition standards/targets for processed foods (PT3)
- Restrict promotion/marketing of unhealthy food (PT4)
- Provide clear/consistent healthy food promotion policy in schools (PT5)

The importance of these policies to Ghana and Kenya was later confirmed when future government action was prioritised by Food-EPI expert panels (n = 34 across Ghana/Kenya), who discussed evidence collected in the respective countries and independently ranked all five areas as priorities for implementation.^12,13^

###  Programme Theories Underlying Policies

Programme theories for the five policy areas are articulated in Box 1.

**Box 1. PT for the Five Policy Areas**

**PT1: Regulate Trade in Goods/Services and FDI**

IF Governments introduce policies that seek to regulate trade in goods, trade in services and/or FDI

THEN industry/businesses will make strategic choices that negate the import, production, processing, retail and/or marketing of unhealthy foods

LEADING TO (*i*) Reduced consumption of unhealthy foods and (*ii*) Increased consumption of healthy foods

**PT2: Regulate Food Health/Nutrition Claims**

IF a government restricts inappropriate claims for health made for foods

THEN consumers limit their consumption of misrepresented food

LEADING TO (*i*) Reduced consumption of unhealthy food and (*ii*) Increased consumption of healthy foods

**PT3: Set Food Composition Standards/Targets for Processed Foods**

IF the Government sets targets for composition of processed foods in terms of healthy and unhealthy ingredients

THEN consumers reduce their purchase of unhealthy foods

LEADING TO (*i*) Reduced consumption of unhealthy foods and (*ii*) Increased consumption of healthy foods

**PT4: Restrict Promotion/Marketing of Unhealthy Food**

IF a Government restricts the ability of businesses to promote unhealthy foods

THEN consumers (the public, especially children) will not purchase unhealthy foods

LEADING TO (*i*) Reduced consumption of unhealthy food and (*ii*) Increased consumption of healthy foods

**PT5: Clear/Consistent Healthy Food Promotion Policy in Schools**

IF schools implement clear and consistent policies on healthy food promotion

THEN children will be exposed to healthy foods (eg, fruit/vegetables), which will have a positive impact on their food choices

LEADING TO (*i*) Reduced consumption of unhealthy food and (*ii*) Increased consumption of healthy foods

Abbreviations: FDI, foreign direct investment; PT, Programme Theoriy.

A format was agreed for the initial PT for each policy action (Box 1). This was developed from the above-mentioned stakeholder discussions, the policy review, evidence identified from scoping searches (see Table 2), and discussions across the research team. This involved ‘reality testing’ how policy intent translates into practice, and considered how contextual factors might influence food consumption. Initial agreed programme theories formed the basis for a common framework to be ‘populated’ with evidence addressing: the components of each policy action; contextual factors; potential mechanisms and outcomes. The review was registered with PROSPERO International Prospective Register of Systematic Reviews (2018) (No: CRD42018111034).

**Table 2:** Evidence From Ghana, Kenya and beyond for PTs for All Five Policies

**PT**	**Summary of Evidence Base**	**Supporting Evidence**			
		**Reviews**	**Ghana**	**Kenya**	**Other Countries**
PT1 trade	Review papers (systematic reviews, narrative commentaries) set out generalised potential pathways between government action and food consumption. Primary studies focusing on Ghana and Kenya indirectly relevant, providing insight on topics relating to trade and/or investment. Limited evidence of policy examples to regulate trade in goods of direct relevance. No examples of action to regulate trade in services/FDI. Limited consideration of differential impacts across population/socio-economic groups.	21 reviews^29,31-33,35-37,41-46,49,58,60,67-70^	10 studies ^12,39,50,52,53,57,59,61,71^	7 studies ^13,47,48,51,56,63,72^	11 papers ^30,34,40,54,55,62,64,65,66,73-74^
PT2 nutrition claims/labels	Extensive international coverage of issues relating to food labelling, including front-of-package and back-of-package marketing.^75-88^ Ghana has seven research studies examining effects of food labelling on consumer attitudes and/or behaviour.^89-95^ Kenya has no identified studies of food labelling, with only partially relevant studies on food composition and consumer perceptions.^96-98^ Across both Ghana and Kenya, therefore, data specifically on effect of health claims is comparatively sparse. Experience from South Africa, further advanced along nutrition transition, demonstrates potential for coherent programme of investigation.^84^Overall, studies are of limited rigour, involving surveys and small-scale qualitative research, but Ghanaian studies benefit from contextual relevance. The need for context sensitive research on the effect of health claims is common theme. Consumer knowledge, use and understanding of nutrition labelling has been investigated extensively in international literature.^84^	14 reviews ^75-88^	7 studies ^89-95^	3 studies ^96-98^	22 papers ^99-120^
PT3 food composition	Few studies focus on actions taken by governments in Africa to set targets for composition of processed foods.^122,123^ Regulations aimed at restricting consumption of unhealthy foods,^124^ have resulted in healthier food choices.^123^ Data from Ghana and Kenya on government actions to regulate consumption of unhealthy foods is scant. The FDA of Ghana enforces food labelling on processed pre-packed foods.^90^ We did not find studies related to enforcement of regulations to restrict consumption of unhealthy foods and beverages in Kenya. Lack of studies on food regulations in most African countries may be partly explained by the lack, until recently, of good quality food composition data, particularly Ghana (2012) and Kenya (2018).^125^	7 reviews ^122,124,126-130^	1 study^90^	0 studies	8 papers ^123,125,131-136^
PT4 food marketing	Evidence supporting PT4 included 28 sources^12,13,110,137-169^(mainly global, focused on High Income countries) with two from Ghana^144-145^ and one from Kenya.^146^ In Ghana and Kenya, political will is communicated in national policies, but with little implementation. Systematic reviews relating to the impact of food marketing on children abound; little data exists on the effect of promoting healthy food or healthy foods choices. Mid-range theories suggest pathways between food promotion and food preferences, food choices, food consumption. Few link food promotion and obesity. Very limited evidence from Ghana and Kenya provides background context. Increasing regulatory diversity with industry self-regulatory approaches as a major response, despite accumulating evidence that industry policies are designed to minimise changes to marketing practices with a minimal impact on reducing children’s exposure to unhealthy food marketing.	7 reviews ^110,137-143^	2 studies ^144-145^	1 study ^146^	18 papers ^147-164^
PT5 school food	Literature on issues related to healthy school food environment in Ghana and Kenya is sparse. (Ghana has eight peer reviewed publications,^165-173^ Kenya nine^174-182^). Most evidence is limited to food provision in schools in context of food security. Most studies are surveys with small sample sizes, or are qualitative. Need for robust studies to assess the healthiness of school food environments, the effect policies have on these environments and the implications they have for childhood obesity and NCDs in general.	8 reviews ^137,143,153,163,167,183-185^	8 studies ^165-173^	9 studies ^175-182^	7 papers ^186-193^

Abbreviations: FDI, foreign direct investment; PT, programme theory; FDA, Food and Drugs Authority; NCDs, non-communicable diseases.

For inclusion, sources had to be published in the English language and focus (in whole/part) on one of the five prioritised policy actions within Ghana, Kenya, or comparable African low- and middle-income countries (LMICs); or, within review papers, in LMICs more generally or at a global level. In contrast to other review types, a realist synthesis includes diverse literature types (unless an item does not relate to any of the theory areas).^27^ Because a paper is excluded in one theory area does not automatically require exclusion from other theory areas. Exclusion criteria are explicit; a clear rationale is documented for each excluded article. Consistent with this, our review asked: Is the evidence provided in this theory area ‘good and relevant enough’ to be included (considering issues of sample size, data collection, data analysis, and claims made).^27^

###  Search Strategy, Sources and Screening

A two-stage search was conducted using MEDLINE, Web of Science and Scopus databases. Exploratory literature scoping was conducted on PubMed MEDLINE (April-May 2018) to identify initial PTs 1-5 from within the included sources. The scoping search was used to refine subsequent searches. Follow up iterative, comprehensive searches were carried out to identify studies/reports from Kenya and Ghana, to test the identified theories (Supplementary file 1). Follow-up searches were conducted in June and July 2018, with search updates for reviews completed using MEDLINE and Scopus (May 2020). Supplementary searching using reference list (backward) checking and citation (forward) searches were undertaken to identify additional papers not picked up by database searches.

 One reviewer initially screened titles to eliminate obviously irrelevant references. The remaining titles and abstracts were divided between five reviewers who, after assessing a test set, applied inclusion criteria to all potentially-relevant sources. Decisions on inclusion were recorded in an Excel spreadsheet. A second member of the team reviewed each final selection of sources across the five policy areas to ensure consistency.

###  Data Collection and Analysis

 For each policy area, sub-teams of two-three members extracted text against the evaluative framework (ie, extracting the components of policy action; contextual factors; potential mechanisms and outcomes). The outcome of interest was change in food consumption, but intermediate outcomes were recorded if mentioned. Data extraction was checked by a second member of the review team. Given that the value of risk of bias assessments within realist syntheses is contested, particularly in examining how complex policies operate (compared to the effectiveness of biomedical interventions) we assessed the overall profile of study designs (ie, the distribution of reviews, quantitative or qualitative research studies and other papers) supporting each policy action. This approach to assessing the whole evidence base, not individual included studies, proved most useful. In summary, the team considered three Rs, namely, the ‘relevance’ of each source (ie, its contribution to theory under test); its ‘rigour’ (ie, credible and trustworthy methods); and its richness (ie, the substance, quantitative or qualitative, of its contribution).

Data analysis was iterative. Evidence for each policy area was mapped against the evaluative framework derived from our initial programme theories. Logic models for each policy action were generated from extracted data, illustrating how each might work within the socio-economic and political environments of Ghana and Kenya. Aligned to retroductive approaches, connected Context-Mechanism-Outcome elements are identified and coded within the data.^28^ Sets of these configurations are presented within a Microsoft Excel table to show individual components and their relationships and to help to explain how, and under what circumstances outcomes relating to food consumption are achieved. The previously agreed programme theories were continually revised. We drew on our multi-country (UK, Ghana, Kenya) and multi-disciplinary (public health, nutrition, epidemiology, information science, politics/geography) expertise to discuss and challenge the logic models, revised programme theories and supporting evidence at regular team meetings. This added analytical rigour. The logic model for each PT was considered in turn, before combining these into a summary conceptual map by consensus.

## Results

**Figure 1:**
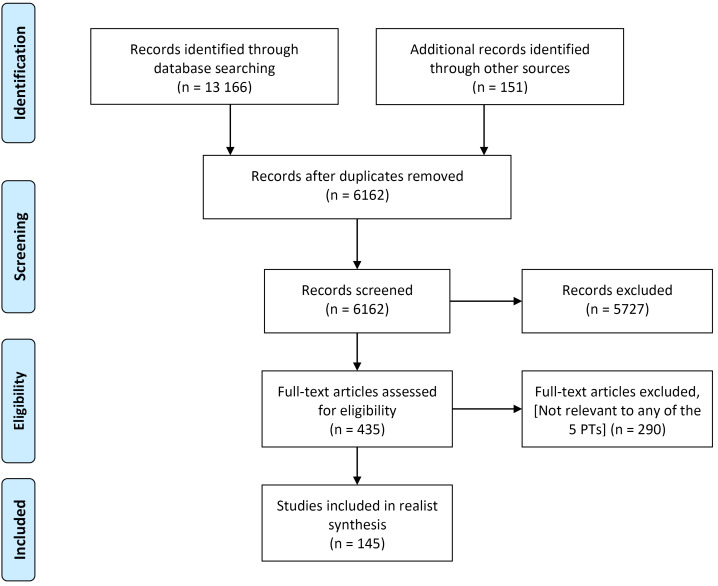
PRISMA Diagram. Abbreviations: PRISMA, Preferred Reporting Items for Systematic Reviews and Meta-Analyses; PTs, Programme Theories.

Overall, 13317 references were retrieved. After removal of duplicates 6162 were assessed for eligibility. Of these, 5727 were excluded at first screening (ie, historical, not human studies, pre-dating included time period) (Figure 1). The remaining 435 were assessed for relevance. Included sources comprised: 46 evidence reviews, 40 primary research studies (Ghana 25 and Kenya 15) and 59 discussion/opinion papers (Table 2).

###  Results From the Empirical Evidence

This section highlights findings for the five focal policy actions; discussing how each could support policy action to improve healthier food consumption in urban Africa, drawing on evidence from Ghana and Kenya to contextualise the findings identified (Table 2).

###  Programme Theory 1: Regulate Trade in Goods/Services and Foreign Direct Investment 

**Figure 2:**
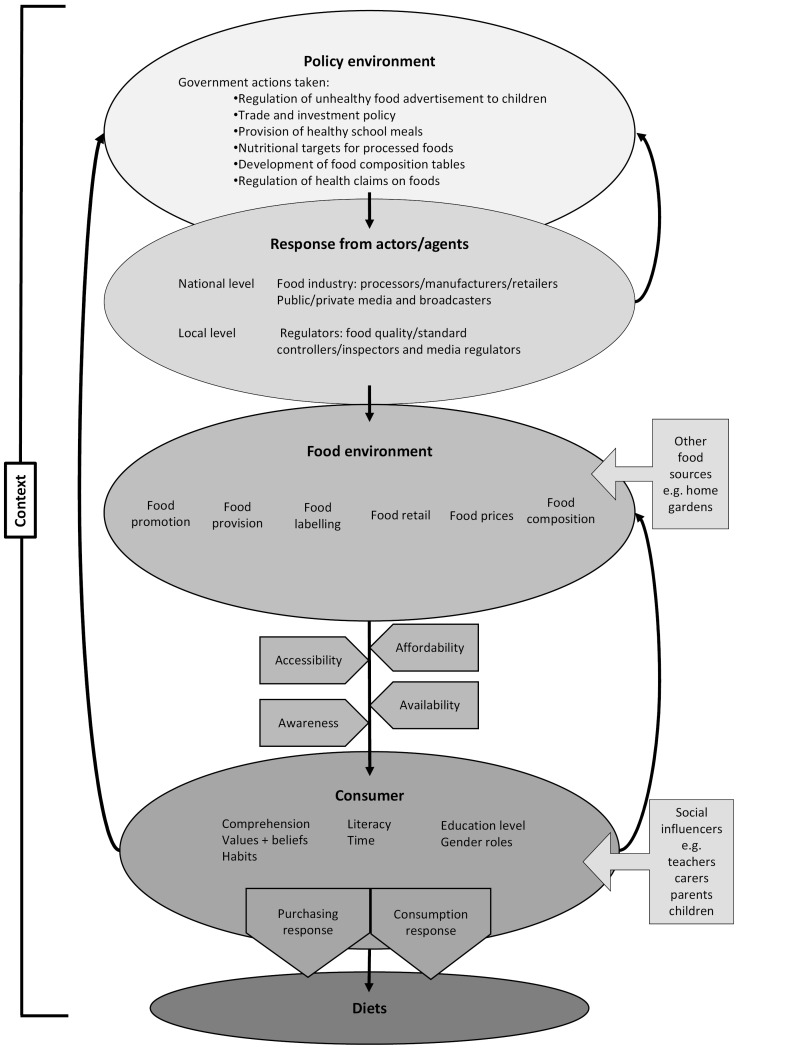
Synthesised Conceptual Map Across Five PTs. Abbreviation: PTs, Programme Theories.

PT1 articulates a generalised pathway between trade and investment policy and food consumption (Table 2) (Supplementary file 2). Primary studies on Ghana/Kenya were indirectly relevant; providing insight on topics relating to trade and FDI. We identified limited policy examples of regulating trade in goods of relevance to urban Africa, no specific examples of intervention to regulate trade in services/FDI, and limited consideration of differential impacts across socio-economic groups. Despite these limitations, we found that trade regulation is assumed to work by providing behavioural incentives/disincentives for stakeholders (eg, international food companies, suppliers, investors, buyers) operating within global, regional and domestic food systems: preventing or encouraging particular forms of trade/investment activity or strategic choices to be made.^29,30^ This, in turn, is assumed to precipitate changes in urban consumer food environments, altering availability, accessibility, desirability, purchasing and consumption of ‘healthy’ or ‘unhealthy’ foods (as highlighted in the summary model (Figure 2).^31-37^

Import barriers (eg, product bans, tariffs, standards) exemplify regulatory actions that theoretically work by disincentivising trade in goods: reducing cross-border flows of ‘unhealthy’ foods to urban centres by preventing/limiting trade in these foods, raising trade costs and/or encouraging ‘healthier’ product reformulation. Included sources highlighted Ghana’s ‘innovative’ standards for meat products to address rising imports of low-quality fatty meat.^38,39^ Regulatory controls on nutrition labelling (see PT2) exemplify trade policies that create ‘technical barriers’ to cross-border movement of packaged foods.^40^ Governments can also regulate trade in services and FDI; for example, introducing diet- or health-oriented conditionalities on FDI by transnational food companies within an integrated package that covers marketing and provision of information (see also PT4).^41^

Removing barriers to trade in goods/services or FDI can theoretically improve healthy food consumption.^31,33,42,43^ Reducing import restrictions (eg, eliminating quotas, removing licensing, reducing tariffs) can incentivise trade in raw materials and finished products, bringing cheaper international food imports (eg, essential foodstuffs, processed food) into domestic markets, increasing food availability, price competition, affordability, and healthy food consumption.^31,32,35,44^ Similarly, removing restrictions on market access for foreign investors in domestic service sectors (ie, retail/supermarkets, marketing) and/or reducing restrictions on foreign ownership of companies, can potentially deliver urban dietary gains: enabling food processing, expansion of food retailing, widening food choices and lowering prices.^35,41,45^ Many LMIC governments, including in Kenya/Ghana, implemented liberalisation measures from the mid-1980s: removing price controls on domestic products like maize and sugar in Kenya, removing import licensing, and loosening investment rules.^41,44,46-48^

The effects of trade policy on healthy food consumption and on the wider food system are strongly mediated by global, regional, national and local contexts.^29,31-33,36,38,41,42,44-46,49^ For trade/investment regulations to work, stakeholders must respond in anticipated ways to incentives/disincentives^29^ and urban consumers must adapt purchasing/consumption behaviours in anticipated ways to food environment changes.^44,48,50^ In practice, many factors affect ‘success’ at these two points and it is difficult to anticipate what will happen in urban Africa due to limited knowledge of the complex socio-material dynamics of supply, demand and local competition, and in contexts occupied by many small and medium enterprises.^43,45,50-53^ Many LMICs collate limited data to inform/evaluate policy action (eg, product-specific retail sales, consumption patterns, sector-specific FDI, market concentration of transnational corporations, existing tariffs/import duties).^33,54^ Effects of trade regulation on urban food consumption *via*, for example, import barrier removal not only depend on how domestic producers respond to new/cheaper products competing in local markets, but also on associated shifts in food environments, food culture and behaviours.^31,33,35,44^ Domestic producers could be displaced and/or domestic staples production converted to export cash crops, leading to reduced availability of traditional crops and increasing people’s exposure to ‘unhealthy’ imported food.^32-35,44^ Impacts on ‘healthy’ consumption depend on types of foods imported (eg, wheat; dairy; highly-processed nutrient-poor), competitive market dynamics and shifts in consumer responses over time.^31,33,35,44^ Included sources offered limited consideration of *who* might be affected by government action, though emphasised differential impacts across urban population groups, with the most vulnerable often disproportionately affected in negative ways.^43,55-58^

While trade liberalisation measures potentially decrease food importation costs, increasing food diversity and availability/affordability of ‘healthy’ foods (eg, staples like maize)^45,47,56^ one review reported mixed results for ‘trade openness’ and undernutrition: with evidence of reductions in underweight and increases in nutrient supply, intakes, and proxies for dietary quality.^43^ Yet lower tariffs have increased imports of sugar-sweetened beverages^58^and may also coincide with increased availability (and likely consumption) of ‘hazardous products’ like sweeteners via a ‘hazard substitution’ pathway.^55^ Kenya’s 2015 removal of sugar protections surfaced fears of cheaper sugar imports competing domestically, making greater quantities of confectionary available.^48^ Growth of vegetable oil imports in Kenya coincided with liberalising reforms, with vegetable fats now commonly consumed by Kenyan women.^48^ FDI is the ‘preferred method’ for transnational companies to enter new markets for processed foods: allowing product marketing that creates demand while adapting to consumer preferences.^41,43^ Evidence highlighted FDI’s role in exposing populations to unhealthy products (eg, sugary, highly-processed foods, soft drinks) in LMICs, and undermining food security in Ghana.^41,43,59,60^

Stricter trade regulation can lead to reduced imports of ‘unhealthy’ food products. Higher tariffs on sugar-sweetened beverages can significantly decrease per capita imports.^58^ In Ghana, standards for meat products reduced imports and availability of low-quality, high-fat meats; with standards more useful than product-specific bans because they apply to imported *and* domestic meats, thus meeting multilateral trade commitments, and automatically apply to new meat products without needing amendments to legislation.^39^ Efforts by Ghanaian producers to get government to impose an additional duty on frozen poultry imports were less successful; tied interests of domestic/international producers/importers and lack of strategic policy framing (eg, as a health/safety issue) undermined such action.^61^

Effective implementation of trade regulation requires government infrastructural capacity, policy framing, coherent/organised alliances/interest groups and public support (potentially with more politically-powerful urban consumers).^12,13,43,61^The complexity of trade-nutrition-health links, makes collaborative capacity between ministries important: promoting understanding and policy coherence, so economic objectives do not undermine ‘preventative’ action on trade and FDI.^40-42,45,46,62^ Achieving this can be challenging in LMICs where liberal economic policies may be stated priorities of government and partner international financial institutions.^31,56,61,63^ In Ghana, trade and health ministries collaborated successfully to control fatty meat imports^39,40^ but found enforcement challenging due to limited local authority capacities.^39,40,53,64^ A key challenge to regulation of FDI is that action may undermine country sources of external financing.^41^ Another challenge is regional trading dynamics, with no evidence identified on how membership of regional trading areas affected action in Ghana and Kenya. However, sources illustrated that treaties can limit ‘policy space’ to intervene, given that regulation of food labelling/composition/marketing may infringe agreements (risking legal challenge) whilst also incentivising trade in ‘unhealthy’ products.^33,42,55,62,65-69^ For example, South Africa’s regional ‘hub’ role promoted import, investment in and access to soft drinks and processed foods in other Southern African Development Community countries.^46,56^

###  Programme Theory 2: Regulate Food Health/Nutrition Claims

PT2 embodies an assumption that providing information on nutritional/health advantages of particular foods/nutrients to consumers suffices to advance healthy food consumption choices (Supplementary file 3). Conversely, inappropriately connecting a food, food component or nutrient to desired health may counter public health objectives.^103^ Seven studies from Ghana examine how food labelling impacts upon consumer attitudes/behaviour,^89-95^ with three studies identified from Kenya (Table 2).^96-98^ Notwithstanding an extensive international literature,^77-87,111^the need for more *context-sensitive research* recurs across included papers. Evidence suggests an asymmetrical relationship; the balance favouring inappropriate claims unless regulated by government. The regulatory environment shapes “the types of food that can and cannot be sold, and who is involved in producing, distributing and selling food”^87^ and seeks to “allow better-informed choice.”^121^However, food labels extend beyond consumer information to offer manufacturers a marketing technique to communicate attributes of products to potential consumers^95^ thereby increasing the likelihood of purchase.^82^

Many countries adopt labelling regulations during the ‘nutrition transition.’^85^ Food labels help regulatory bodies to protect public interests by ensuring that food produced and sold meets required standards.^95^ Food labels also remind citizens of their government’s duty of care when intervening in their daily lives.^95^ Health claims in Ghana are regulated under the Food and Drugs Law, 1992. Kenya provides voluntary state-sponsored guidelines that define nutrients to be listed and on what basis.^98^ Labelling is not mandatory unless a health or nutrition claim is made or unless the food is for special dietary uses. The food industry may respond to tighter control of health claims by launching new products targeting health-conscious consumers. Other consumers persist in an ‘unhealthy/tasty’ intuition, disincentivising firms from reformulating existing products.^86^

For labelling to work, consumers must read, understand, and believe the information content of the label. Nutrition knowledge exerts a low/average impact on consumer food choices^92^ even among those who report reading food labels. Ghanaian consumers are influenced by product price (cost), time (convenience), adverts, label information, expiry data, nutritional information, ingredients, taste and appearance.^89,93^ African studies report that price is paramount when selecting food products, irrespective of quality and nutritional value.^106^ In Kenya, geographical origin may be influential.^96^ Too much information can confuse consumers and too little may mislead them.^110^ Advertising and price,^95^ cultural expectations, the taste-nutrition trade-off,^120^ and availability of affordable and attractive alternatives contend with regulation of health claims to influence purchase and consumption behaviour.^117^ Limited comparison exists with other African countries; Kenya (25%) and Ghana (33.3%) demonstrate higher non-compliance in nutrition claims than South Africa (10%).^105^

Consumers must have time to read and understand health claims.^80^ Although 75/100 consumers in Ghana reported reading labels prior to selecting food^92^ self-reporting is inaccurate.^115^ One Ghanaian study showed that consumers are not dissuaded by a label in a language they could not understand.^89^ A contemporaneous study found low levels of label reading.^91^ Even Ghanaian respondents with tertiary education experience difficulties explaining recommended dietary allowances per serving.^92^ Older individuals, white participants and those with higher education and income read nutrition information more frequently.^107,109,119^ Females were most likely to read^91^ and be influenced by food labels.^93^

Studies link reading a label that advances a health claim to consumption of that product. A meta-analysis found that perceptions of health claims varied based on the food involved and how it was perceived before the health claim.^79^ Consumers may read information to assess nutritional value or health properties, to avoid allergens and to determine quality.^106^ Consumers reading front-of-pack claims may conclude a product is healthier, making them more likely to purchase, particularly if predisposed towards that product.^80^ Combining short front-of-pack health claims with full claims on the back of the package can lead consumers to better process and believe the claim.^110^Commentators suggest that improving educational levels in urban centres can be exploited as the population becomes interested in food labelling.^75^ For this type of action to work, consumers need to be educated to improve nutritional knowledge and to utilize information on food labels. Food labels often require simplification^109^; simple terminology, pictures/colours, a single health endorsement logo and bigger fonts.^107^ Presentation of health claims may favourably impact upon product, nutrition attitudes, and purchase intentions.^118^ However, price^106^ and ingrained purchasing habits exert greater influence than the label.

###  Programme Theory 3: Set Food Composition Standards/Targets for Processed Foods 

PT3 postulates that, “if governments set targets for composition of processed foods, in terms of healthy and unhealthy ingredients, consumers will reduce their purchase of unhealthy foods and this will ultimately lead to (*i*) reduced intake of unhealthy food and (*ii*) increased consumption of healthy foods” (Supplementary file 4). The theory statement was informed by existing evidence from high-income countries demonstrating that reformulating processed foods could significantly contribute to reducing the consumption of unhealthy foods and beverages to reduce the risks of diet-related NCDs.^135^ This review found few studies conducted in Ghana/Kenya or in other African countries (Table 2) focusing on actions taken by governments towards the setting of targets for composition of processed foods, in terms of healthy and unhealthy ingredients. Limited studies from South Africa^122,124,126-127^ report that regulations have been legislated and implemented by the South African government, aimed at restricting the promotion and sale of unhealthy processed foods. In Kenya, we found no studies specifically reporting on actions by the government to regulate sales or consumption of processed unhealthy foods. One small-scale study^90^ mentions that the Food and Drugs Authority (FDA) of Ghana enforces food labelling on all processed pre-packed foods.

The lack of targets set, or general non-existence of clear guidelines/standards in many African countries to facilitate the regulation of sales and consumption of processed unhealthy foods and beverages could be widely impacted by poor quality or non-existent food composition data.^128^ Experts warn that low/poor quality food composition tables can misdirect or lead to inappropriate policies for the regulation of unhealthy food purchase or their consumption.^125,128^ Conventionally, high-quality food composition tables are recommended as useful resources that can be used to facilitate the reformulation of processed foods because they provide a better standard for evaluating the effects of diets for individuals and populations.^134,135^

In high-income and LMICs, some evidence supports the important role of high quality food composition data in national food standards guidelines development and implementation to regulate processed unhealthy foods and sugar-sweetened beverages, and the impact of this in reducing diet-related NCDs burden.^134,135^For instance, in South Africa, food composition tables make it possible for government to enact legislation related to advertising, labelling, and consumption of certain food items (See PT2). This aims to ensure that consumers are provided with adequate useful nutritional, compositional and other information related to food products manufactured locally or imported and sold in South Africa.^122^ This has impacted positively on changes in consumers’ behaviours towards healthier food choices and preventing food waste.^122,126,129^

In African countries that lack high quality food composition databases to provide accurate estimates of nutrients for local food items, such restrictions barely exist. For instance, in Uganda, the lack of a national food composition database is suggested as a potential reason for the lack of national dietary guidelines.^131^ Lack of expertise and skills of African nutrition scientists, as well as capacities of governments to collect and undertake food/nutrient analysis, are identified as key factors influencing food composition data quality, and/or lack of food composition tables in setting targets.^126,132^ The West African Food Composition Table, including local ingredients from Ghana, provides an opportunity to regulate the sales of processed foods,^136^ and in Kenya, national food composition tables have recently been published.^133^ However, no such regulation has yet taken place in either country.

###  Programme Theory 4: Restrict Promotion/Marketing of Unhealthy food 

PT4 hypothesizes that if governments introduce policy measures that restrict marketing of unhealthy foods, their production, processing, importation, marketing by industry will reduce–leading to reduced consumption (Supplementary file 5). Plentiful evidence (Table 2) shows that food marketing affects food preferences, purchase behaviour, and pester power/purchase requests (of children).^103,137,138,142,149,153,156,160^Independent of other factors, exposure to unhealthy food marketing is also a modifiable risk factor for obesity.^163^ As a result, the World Health Organization (WHO) through World Health Assembly (WHA) Resolution 63.14 called for governments to ensure that “… settings where children gather are free from all forms of marketing of unhealthy foods (eg, foods high in saturated fats, trans-fatty acids, sugars or salt).”^151^ Ghana and Kenya are both signatories.

Globally, countries are actively implementing WHA Resolution 63.14, although few are from Africa.^164^ Only Morocco has fully achieved implementation of NCD progress monitoring indicator #7C “marketing to children restrictions.”^164^ In Ghana and Kenya, government policies exist to restrict exposure and power of promotion of unhealthy foods to or for children across diverse settings. The Ghana FDA requires products to be registered, and advertisement scripts approved before they can be advertised.^150^ National NCD Policy priorities to achieve healthy diets for Ghana include regulating advertising of unhealthy foods and non-alcoholic beverages, particularly to children.^20,158^ In Kenya, the Code of Advertising Practice and Direct Marketing (2003) restricts advertisements addressed to or targeted at children with potential to harm them mentally, morally, physically or emotionally.^147^ The Kenyan National School Health Policy (2017) prohibits marketing foods and beverages within and around schools.^176^ Both Ghana and Kenya embed policies within the national NCD prevention and control strategies focusing on restriction of unhealthy food promotion to children in schools, however local policy-makers rated implementation as low.^12,13^

Recent research confirms inadequate government action in enforcing the policies.^12,13^ Unhealthy foods are sold to Ghanaian children in school by private/independent vendors^170^ and soft drinks are heavily advertised.^38,152^ Contextual evidence from beyond Ghana and Kenya demonstrates that incentives (positive or negative), and underlying factors such as food security, food cost, food/nutrition literacy, health literacy and the respective political economies are important.^89,92,93,155,157,159^Effective implementation and enforcement of these interventions requires robust planning processes and resources.^161^ Additionally, all stakeholders (government and non-government) should respect the core values of the intervention. Also critical is the mode of regulation; governments favour approaches that encourage the food industry to self-regulate,^160^ notwithstanding accumulating evidence that industry policies have minimal impact on reducing children’s exposure to unhealthy food marketing.^139,142,148,162^ In Ghana and Kenya, political will is communicated in national policies, but with insufficient implementation. While systematic review evidence relating to the impact of food marketing on children is plentiful, it fails to address the effect of promoting healthy food on healthy food consumption.

###  Programme Theory 5: Clear/Consistent Healthy Food Promotion Policy in Schools 

Literature is sparse on school food environments in Ghana/Kenya (Table 2). Evidence focuses on school food provision to ensure food security in marginalised populations to increase enrolment in school and improve school grades. Most studies are surveys with small sample sizes or are qualitative in nature. PT5 postulates that if government regulates the school food environment, then healthy foods will be provided or sold around schools, reducing/limiting children’s exposure to unhealthy foods (Supplementary file 6). The school food environment exerts a large impact on the dietary intake of children and adolescents, contributing between 19%-50% of daily calories.^190^ Food and beverages consumed at school are typically available through meals provided/sold by school/around school or brought to school. A whole school policy should therefore target all avenues that increase access to unhealthy foods as well as encouraging healthier food choices.^191^ School food policies can promote healthy food consumption through several means: *nutrition guidelines*- providing nutrition standards for menu planning based on food and/or nutrients and applied at school meal programs or at other meals sold in school environment; *regulation of food and beverage availability*- by controlling the type of food and beverages sold or provided at school; *price intervention*- free or subsidised provision of healthy foods, or controlling the price of foods or beverages sold in schools. A systematic review of school food policies globally showed that nutrition guidelines and price interventions focused on healthier foods are effective in improving the school food environment and dietary intake.^184^

Effective, enforced school food policies require school-level implementation; Ghana and Kenya have successfully used a decentralized approach.^186,187^ Where schools lack a functional implementation committee, such as in some regions in Ghana, implementation is a challenge.^173^ Linking school feeding policies directly with agricultural development in communities surrounding schools, can lead to success in implementing policies,^189^ incentivising farmers to grow healthier foods and thus sustaining local food systems.

To be successful, government at all levels need resources for implementing and evaluating policies. Programmes are planned at national level, with local authorities/councils and municipalities responsible for implementation. In some cases, parents take responsibility for preparing meals.^171^ The Ghana school feeding programme relies on financial and technical support from government and development partners; partnerships with the Ministry of Agriculture are needed to ensure that local products are used.^188^ In Kenya, a national policy framework for school feeding programs exists, although national school feeding guidelines are not established. School feeding in Kenya lacks stable funding and the institutional and implementation arrangements necessary for sustainability and efficiency.^187^ Success of school policies also depends on appropriate monitoring. For example, in Kenya, an innovative approach was the development of a computer-based monitoring system jointly run by the Ministry of Education and World Food Programme, identifying poor management practices and assistance needs in vulnerable areas.^178^ Overall, school feeding programme targeting and implementation are best undertaken at school, rather than, national level. Most LMICs do not have standalone policies promoting healthy food consumption in schools for preventing NR-NCDs and instead are established to increase school attendance and address food security of vulnerable populations; thus, emphasizing food supply (quantity) rather than healthy food consumption (quality). For example, Ghana piloted and scaled-up the Ghana School feeding programme to encourage educational participation and improve nutrient intake^167,169,172^; while Kenya targeted socially-disadvantaged and nutritionally-vulnerable children, used as an incentive to attract school-aged children to class and was largely supported by World Food Program funding but transitioned to a government funded program in 2009.^174,180^

Positive elements in guidelines to provide healthy food consumption include micronutrient fortification of commonly-eaten foods and school education to increase children’s knowledge of healthy dietary behaviours.^169,172^ Other programmes promote healthy diets to teachers and parents, regulate products sold in school canteens and by neighbourhood vendors and improve dietary knowledge,^192^ leading to higher nutrient intakes and dietary diversity,^165,169,172,181^ and increased vegetable consumption in families with recipient children.^182^ Consequently, overall health outcomes can be better among children in schools where school feeding programs are implemented.

Although the main school-based food policies in Ghana and Kenya involve school feeding programs, unhealthy foods are frequently available. More than half of Ghanaian schools sell food, most of which is unhealthy^170^ and private food vendors frequently sell unhealthy food adjacent to school^166,193^ exposing children to energy dense foods.^165^ In both Ghana and Kenya, children who take money or food to school often purchase energy-dense nutrient poor foods.^166,175^ In Ghana, the national policy for prevention and control of NCDs recommends restriction of fizzy drinks,^145,168^ however this has not served to curb vendors from selling unhealthy foods and drinks to schoolchildren.^170^ Evidence suggests that school food policies may support changes to the order of foods presented in buffets which may, in turn, encourage shifts towards healthier consumption.^110^ However, in African contexts, school staff and economic dynamics influence behaviours.

## Discussion and Conclusion

This review examined policy actions to support healthier food consumption in urban Africa, with Ghana and Kenya as focal examples to contextualise what policy actions might work to prevent NR-NCDs, for whom, and under what circumstances. Taken together, findings provide a novel and comprehensive evaluation of how five types of government action, identified as particularly important for our selected African countries, may exert effects. We found evidence that implementing these five policies could positively influence the healthiness of food environments and consumption in urban Africa. However, the drivers of (un)healthy food environments and consumption are clearly shaped by a complex interplay of economic, social and political drivers acting at a range of geographical scales. Stakeholders at all levels (local, national and global) have differing roles to play in ensuring healthy food environments and consumption in urban Africa. Our contribution adds to existing academic work that seeks to understand how food policies work, by more fully considering the context of urban Africa and the “nutrition transition.”^194^

###  Social, Political and Economic Complexities in Practice

The five focal PTs suggest relatively simple causal pathways between intervention and effects; with success predicated on assumed predictable and rational choices of stakeholders to differing forms of state intervention. Yet our findings illustrate the socio-cultural, economic and political complexity of policy action in practice, revealing whole system impediments to compliance and illustrate the interdependence of policies seeking to effect transformational preventative action within African food systems. For example, trade and investment regulations (PT1) are inevitably enacted (or not) against a politically and economically contingent backdrop. Intersecting with trade, nutrition labelling, as explored through regulation of food claims (PT2) and setting targets for food composition (PT3), is similarly complex. While in theory the aim is to “induce a food-systems response; overcome barriers to meeting healthy preferences caused by inadequate information,”^194^ the contextual reality of urban Africa sees Governments influenced by competing actors, including industry actors, leading to poor enactment and enforcement.^195,196^ Where policies exist and are enforced, outcomes can be suboptimal due to a myriad of contextual and sociocultural influences. For instance, where textual labelling is in English, functional literacy and health literacy are required, yet these are low in urban Africa.^197^ Additionally, literate, but busy urban consumers lack time to read and process information. Similarly, school food policies (PT5) seek to “overcome barriers to meeting healthy preferences caused by lack of financial or physical access…and poor information skills.”^194^ Elsewhere, evidence suggests that school food policies may encourage children to reassess their preferences at point-of-purchase.^110^ However, in African contexts, school staff and economic dynamics influence behaviours.

Integration of the different programme theories (Figure 2) highlights that the five policies target interfaces between food environment and consumers, within the wider urban African food system, through the enabling (or disabling) forces of food accessibility, affordability and availability. These in turn are influenced by the consumer’s relationships with the retail sector in urban Africa, as well as by political and economic drivers, such as food price volatility and globalized trade. Laws and regulations are key to implementing all five policy actions, whether by implementing national regulations around food composition and health claims (usually required by an FDA), controls on the media for marketing of unhealthy food and beverages by national government, and regulations on trade and FDI. Controlling food sold in/around schools requires proactive regulation, set at national-level, to ensure implementation by local and municipal authorities and other actors. The convenience of food is a further mediator of food consumption, driven by changing dietary habits and (lack of) time, often leading to increased consumption of energy dense foods and beverages. The increasing importance of convenience is shaped by wider demographic drivers linked to rapid urbanisation and rural to urban migration, as well as sociocultural drivers, such as women’s empowerment. Consumers’ awareness of healthy diets is an important mechanism for mediating food consumption through comprehension, education, literacy and beliefs.

###  A Need for Better Data-Evaluation Systems

Contextualised Food Composition Data are key to how policies operate, for example in controlling marketing to children or food labelling, and could explain contextual differences between Ghana and Kenya. Yet, such data are either unavailable, poor in quality of or dated. Food Composition Tables specific to Ghana have not been updated since 1975 with Ghana relying, instead, on regional West African Food Composition Tables^136^ while Kenya has recently launched its own locally developed food composition tables.^133^ Significantly, all programme theories lacked other evidence directly relating to urban African contexts. Our findings thus highlight a need for better data systems to inform and monitor transformational policy action. Research, monitoring and evaluation systems are critical to government policy infrastructure for LMICs, including Ghana and Kenya, for effective action within food systems to address NCDs.^12^

###  Dealing with Interdependencies between Policy Actions

Interdependencies between policy actions (eg, between regulatory action on trade/investment and nutritional labelling), and the critical role of contextualised food composition data, highlight that transformational policy action for urban contexts in Africa depends on the complex interplay of economic, political and socio-cultural forces. These interdependencies illustrate the need for policy coherence; particularly challenging in African contexts where ‘policy space’ to address healthy food consumption is limited by the prioritisation of economic growth by governments and partner IFIs, the role/interests of economic stakeholders in external financing and policy decision-making, and limits in government infrastructural capacities.^12,13^ An additional challenge is the persistence of food insecurity impacting on government policy decisions on the promotion of healthy foods consumption in Africa.^12,13^Therefore, there is the need to highlight the potential double duty actions of the policies being discussed. These issues are highlighted in relation to trade policy actions, and substantiate studies that find limited institutional checks and balances on commercial policy influence in Ghana^12^ and Kenya,^13^ which can drive resistance to policy implementation. As evidenced in other LMICs, policy action to regulate private sector activity relating to food and nutrition can quickly be revoked following extensive industry lobbying at government-level.^196^ For example, Morocco repealed its Sugar Sweetened Beverage tax in 2018 prior to implementation in 2019 in response to pressures from the agri-food industry.^195^ A year later, an attempt was made to introduce a significantly ‘watered-down’ bill.^198^ Only South Africa within the African Region has been able to introduce such a tax with concerted efforts, resources, and alliances of civil society, academia, and government to defeat resistance from food companies. Implementation of WHA Resolution 63.14 has faced similar challenges.

Of 54 countries in Africa, only Morocco fully achieved implementation of WHO NCD progress monitoring indicator #7C “marketing to children restrictions.”^164^ The political economy of implementing healthy food system actions within the African context is unique and complex. Studying the myriad of influencers (functional literacy, health literacy, data and related resources, power etc), is required to fully appreciate the variegated political economies of the African food system, and to intervene effectively.

###  The Importance of Government Institutional Capacities

Our findings highlight the importance of government institutional capacities here: to develop policy collaboratively across government, to develop alliances with non-government actors, and to implement and enforce decisions at multiple levels. Capacity for enforcing regulatory action was highlighted as important to trade policy action, yet limited by what authorities can achieve within existing resources. Controls on food labelling and food composition similarly require capacity for enforcement as highlighted in previous studies.^12,13^

One such actor is The Scaling Up Nutrition Network (a non-for-profit network of academics and non-academics, including government agencies, United Nations agencies, international organisations, the private sector and civil society groups, that harnesses multiple sectors for effective global and in-country planning and implementation of evidence-based nutrition policies and programmes). This Network may have a potential role to play; in supporting cross-sectoral alliances, in better understanding local contexts and in framing strategic action to prioritise NR-NCDs.^157^

###  Strengths and Limitations

 Key strengths of this realist review include an integrated approach that draws on systematic reviews, primary data and the involvement of high-income and LMIC stakeholders. However, such a synthesis is limited by the evidence available for inclusion. Much of the data was indirect; ie, the research questions of sources did not always directly correspond with the focal programme theories. Findings from the two focus countries may not be applicable internationally. Additionally, many studies employed weak research designs potentially impacting on the results. Lastly, in synthesising the current evidence base, the realist review highlights the dearth of country-specific information on how and why policy actions might ‘work’ (or not). Results, although indicative of the available evidence, should be viewed cautiously until directly-relevant studies become more plentiful.

## Ethical issues

 Not applicable.

## Competing interests

 AB holds an honorary contract with the WHO. MH holds an honorary contract with the University of Sheffield. No other relationships or activities have been disclosed.

## Authors’ contributions

 AB and MH are both responsible for the study design. AB performed the searches and led data extraction and analysis. All Authors selected items for inclusion, extracted data, checked data extraction and contributed to data analysis. All authors contributed to writing the manuscript.

## Disclaimer

 The views presented in this paper are those of the author(s) and do not necessarily represent the views of the funder or of their employing organizations.

## Funding

 Sources of support: Review undertaken as part of the Dietary Transitions in African Cities (TACLED), involving a collaboration between the University of Ghana, African Health and Population Centre, Kenya; and the Universities of Sheffield, Liverpool, Loughborough in the UK. The TACLED project was supported by a Global Challenges Research Fund Foundation Award from the UK MRC [grant number MR/P025153/1], and supported by AHRC, BBSRC, ESRC and NERC. The funders played no role in the design of the study, data collection, data analysis, interpretation of the data or writing of the publication.

## Authors’ affiliations

^1^School of Health and Related Research (ScHARR), University of Sheffield, Sheffield, UK. ^2^School of Public Health, University of Ghana, Accra, Ghana. ^3^Institute for Health and Society, Newcastle University, Newcastle upon Tyne, UK. ^4^African Population and Health Research Center (APHRC), Nairobi, Kenya. ^5^French National Research Institute for Sustainable Development (IRD), NUTRIPASS Unit, IRD-Univ Montpellier, Montpellier, France.

## Supplementary files


Supplementary file 1. Search Strategy.

Supplementary file 2. Pathway From Trade/Investment Policy to Food Consumption.

Supplementary file 3. Pathway From Nutritional/Health Information to Healthy Food Consumption.

Supplementary file 4. Pathway From Food Composition Standards to Healthy Food Consumption.

Supplementary file 5. Pathway From Restricting Promotion/Marketing of Unhealthy Foods to Reduced Consumption of Unhealthy Foods.

Supplementary file 6. Pathway From Food Promotion/Regulation in Schools to Reduced Consumption of Unhealthy Foods.
