# Supplementary material for: Policy Action Within Urban African Food Systems to Promote Healthy Food Consumption: A Realist Synthesis in Ghana and Kenya
Source: Int J Health Policy Manag. 2021 Feb 9;10(12):828–44. doi: 10.34172/ijhpm.2020.255 (PMC9309963; doi:10.34172/ijhpm.2020.255)
Supplement: Supplementary file 1 — Search Strategy. [file ijhpm-10-828-s001.pdf]

Supplementary file 1. Search Strategy

Topic area and search terms
1. Nutrition
Food* OR Feed* OR Diet* OR Nutrition*
2. Geographical Location
Ghana or Ghanaian or Kenya or Kenyan or Africa or African
3. Limits
English only, 2000-2019; 2000-2020 (for reviews)
