# Supplementary figures and images for: Policy Action Within Urban African Food Systems to Promote Healthy Food Consumption: A Realist Synthesis in Ghana and Kenya
Source: Int J Health Policy Manag. 2021 Feb 9;10(12):828–44. doi: 10.34172/ijhpm.2020.255 (PMC9309963; doi:10.34172/ijhpm.2020.255)

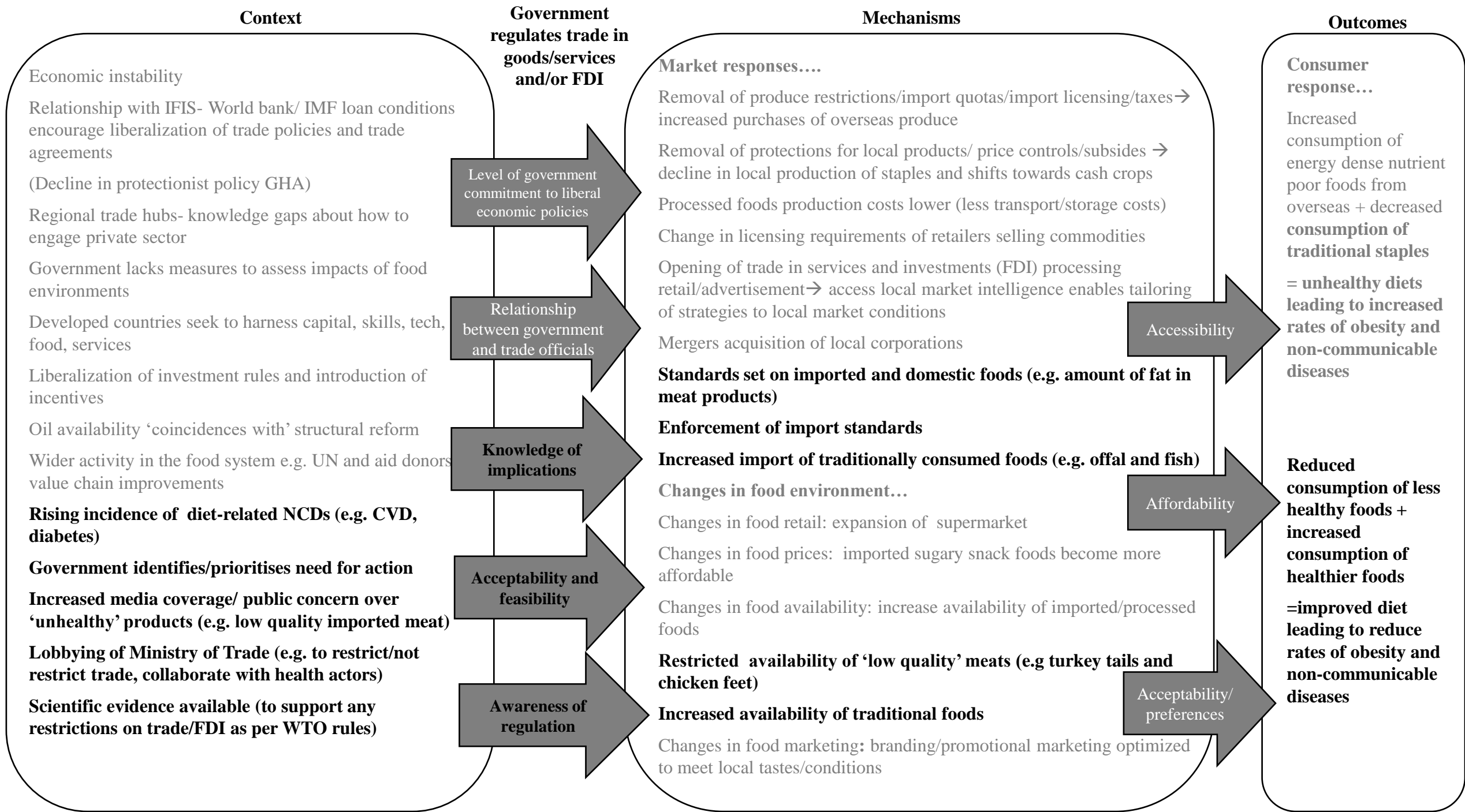

Supplement: Supplementary file 2 — Pathway From Trade/Investment Policy to Food Consumption. [file ijhpm-10-828-s002.pdf]
