# Supplementary material for: Policy Action Within Urban African Food Systems to Promote Healthy Food Consumption: A Realist Synthesis in Ghana and Kenya
Source: Int J Health Policy Manag. 2021 Feb 9;10(12):828–44. doi: 10.34172/ijhpm.2020.255 (PMC9309963; doi:10.34172/ijhpm.2020.255)
Supplement: Supplementary file 3 — Pathway From Nutritional/Health Information to Healthy Food Consumption. [file ijhpm-10-828-s003.pdf]

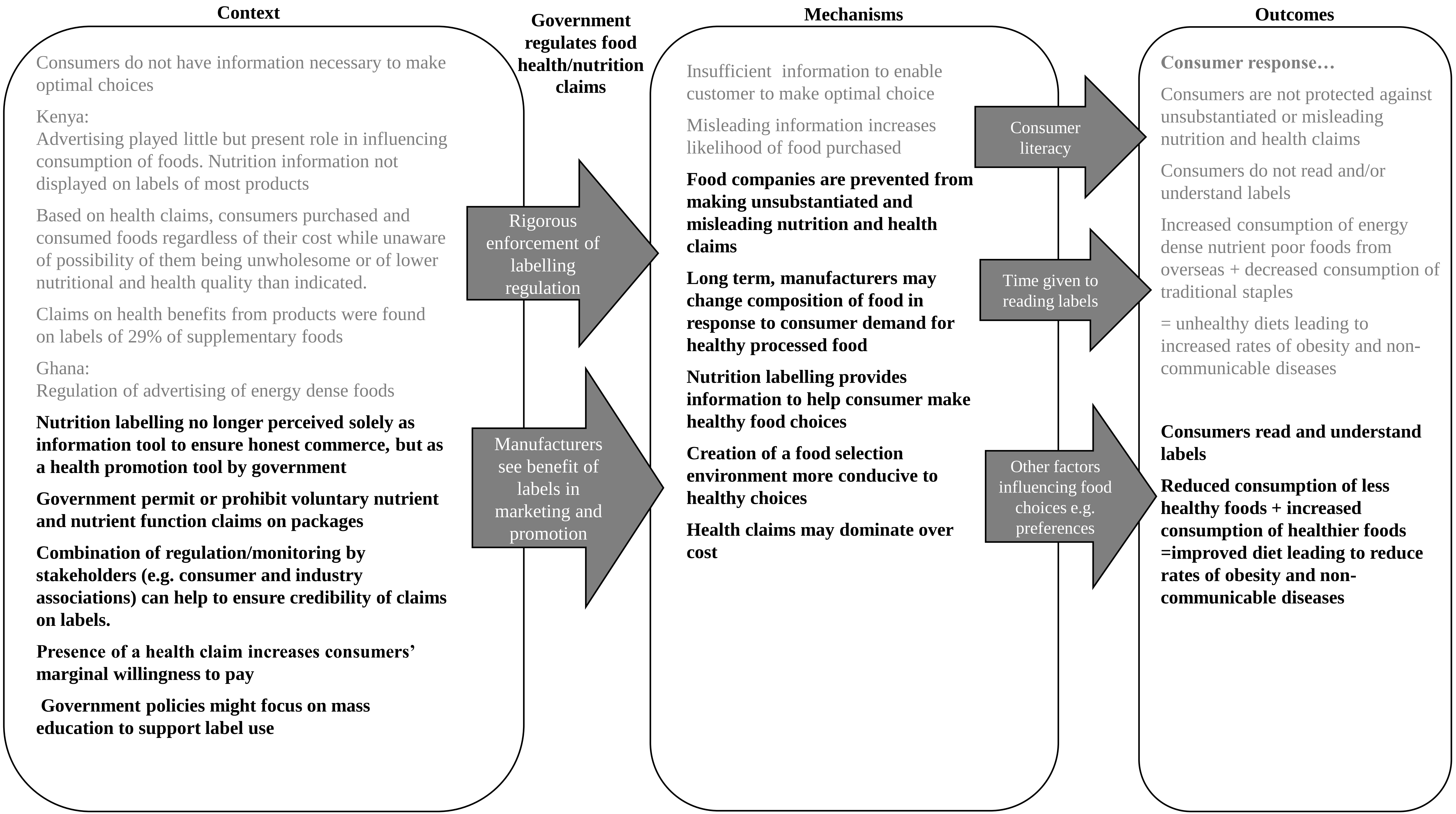
Context
Consumers do not have information necessary to make optimal choices
Kenya:
Advertising played little but present role in influencing consumption of foods. Nutrition information not displayed on labels of most products
Based on health claims, consumers purchased and consumed foods regardless of their cost while unaware of possibility of them being unwholesome or of lower nutritional and health quality than indicated.
Claims on health benefits from products were found on labels of 29% of supplementary foods
Ghana:
Regulation of advertising of energy dense foods
Nutrition labelling no longer perceived solely as information tool to ensure honest commerce, but as a health promotion tool by government
Government permit or prohibit voluntary nutrient and nutrient function claims on packages
Combination of regulation/monitoring by stakeholders (e.g. consumer and industry associations) can help to ensure credibility of claims on labels.
Presence of a health claim increases consumers' marginal willingness to pay
Government policies might focus on mass education to support label use
Government regulates food health/nutrition claims
Rigorous enforcement of labelling regulation
Manufacturers see benefit of labels in marketing and promotion
Mechanisms
Insufficient information to enable customer to make optimal choice
Misleading information increases likelihood of food purchased
Food companies are prevented from making unsubstantiated and misleading nutrition and health claims
Long term, manufacturers may change composition of food in response to consumer demand for healthy processed food
Nutrition labelling provides information to help consumer make healthy food choices
Creation of a food selection environment more conducive to healthy choices
Health claims may dominate over cost
Consumer literacy
Time given to reading labels
Other factors influencing food choices e.g. preferences
Outcomes
Consumer response…
Consumers are not protected against unsubstantiated or misleading nutrition and health claims
Consumers do not read and/or understand labels
Increased consumption of energy dense nutrient poor foods from overseas + decreased consumption of traditional staples
= unhealthy diets leading to increased rates of obesity and non-communicable diseases
Consumers read and understand labels
Reduced consumption of less healthy foods + increased consumption of healthier foods =improved diet leading to reduce rates of obesity and non-communicable diseases
