# Supplementary material for: Policy Action Within Urban African Food Systems to Promote Healthy Food Consumption: A Realist Synthesis in Ghana and Kenya
Source: Int J Health Policy Manag. 2021 Feb 9;10(12):828–44. doi: 10.34172/ijhpm.2020.255 (PMC9309963; doi:10.34172/ijhpm.2020.255)
Supplement: Supplementary file 4 — Pathway From Food Composition Standards to Healthy Food Consumption. [file ijhpm-10-828-s004.pdf]

Context

Few African countries have high quality food composition database/tables that would enable governments to set standards

Many African countries lack capacity to undertake nutrient analysis

No local reference sources exist to allow companies to draw on to inform appropriate food labelling and advertisement

Governments do not recognise the important role food composition data have in influencing food product composition and regulations of unhealthy foods

The African Network of Food Data System (AFROFOODS) is part of the INFOODS network that helps to address the problem of lack of/low quality food composition data in Africa

A global Food-Monitoring Group (FMG) has developed protocols to allow the collection, analysis and standardisation of data on processed foods.

GHA- West African food composition table, which includes Ghanaian foods ingredients, provides opportunity for the government to develop and implement policies to improve food quality

Currently it is the responsibility of the Ghana Food and Drugs Authority (GFDA) to regulate the importation and sales of processed foods that comes into the Ghanaian market, as well as enforce food labelling on all processed pre-packaged foods.

Kenya has recently published its food composition table that could offer an opportunity to improve diet adequacy in the country

Government sets food composition standards/targets for processed foods

Government recognizes advantages of FC data

Government supports working groups to facilitate production of use of FCTs

Mechanisms

Absence of high-quality food data→ misdirect research and nutrition efforts → FCTs not created→ unhealthy processed food not identified→dietary guidelines/labelling not developed

Companies use internationally reputable food composition tables as a reference source to label foods rather than local reference sources--> inaccurate labelling/advertising.

Government efforts to improve diets less effective

Availability of FCTs in (SA) has enabled the development of policies, programmes and legislations to regulate foods production, advertisements, sales and consumption.

Examples of products reformulated/ fortified:
salt reduction in foods
regulation of Trans fats in foods
compulsory iodisation of table salt (SA).

Regulations around nutritional, compositional and other information related to foodstuffs manufactured, imported and sold locally

Regulation/restricting of the consumption of certain foods that are considered unhealthy, as well as the development and implementation of fiscal policy interventions as a mechanism to influence consumer food-purchasing behavior in (SA)

Provision and use of food labels

Use of dietary guidelines

Product availability/ accessibility /affordability

Outcomes

Nutritionally poor processed foods continue to be imported/produced

Consumer response…Customers misinformed about nutritional quality of food choices

Increased consumption of energy dense nutrient poor foods from overseas +

Decreased consumption of traditional staples

= unhealthy diets leading to increased rates of obesity and non-communicable diseases

Consumers provided with accurate nutritional, compositional and other relevant information related to food produced locally or imported.

Food produced/ available has a better nutritional profile for health.

e.g. elimination of iodine deficiency disorders among South African population.

Reduced consumption of less healthy foods +

Increased consumption of healthier foods

=Improved diet leading to reduce rates of obesity and non-communicable diseases
