# Supplementary material for: Policy Action Within Urban African Food Systems to Promote Healthy Food Consumption: A Realist Synthesis in Ghana and Kenya
Source: Int J Health Policy Manag. 2021 Feb 9;10(12):828–44. doi: 10.34172/ijhpm.2020.255 (PMC9309963; doi:10.34172/ijhpm.2020.255)
Supplement: Supplementary file 5 — Pathway From Restricting Promotion/Marketing of Unhealthy Foods to Reduced Consumption of Unhealthy Foods. [file ijhpm-10-828-s005.pdf]

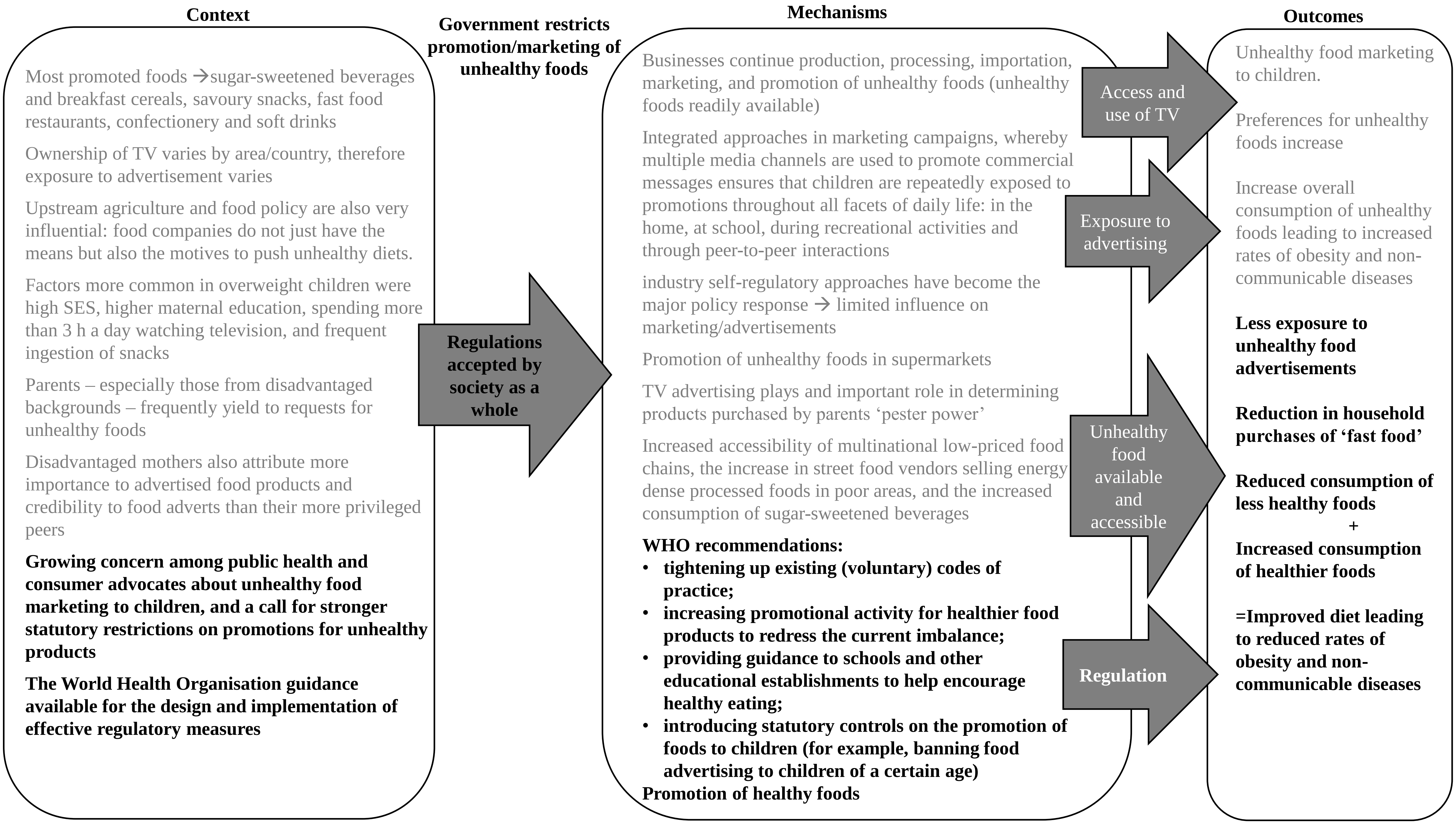
Context
Most promoted foods →sugar-sweetened beverages and breakfast cereals, savoury snacks, fast food restaurants, confectionery and soft drinks
Ownership of TV varies by area/country, therefore exposure to advertisement varies
Upstream agriculture and food policy are also very influential: food companies do not just have the means but also the motives to push unhealthy diets.
Factors more common in overweight children were high SES, higher maternal education, spending more than 3 h a day watching television, and frequent ingestion of snacks
Parents – especially those from disadvantaged backgrounds – frequently yield to requests for unhealthy foods
Disadvantaged mothers also attribute more importance to advertised food products and credibility to food adverts than their more privileged peers
Growing concern among public health and consumer advocates about unhealthy food marketing to children, and a call for stronger statutory restrictions on promotions for unhealthy products
The World Health Organisation guidance available for the design and implementation of effective regulatory measures
Government restricts promotion/marketing of unhealthy foods
Regulations accepted by society as a whole
Mechanisms
Businesses continue production, processing, importation, marketing, and promotion of unhealthy foods (unhealthy foods readily available)
Integrated approaches in marketing campaigns, whereby multiple media channels are used to promote commercial messages ensures that children are repeatedly exposed to promotions throughout all facets of daily life: in the home, at school, during recreational activities and through peer-to-peer interactions
industry self-regulatory approaches have become the major policy response → limited influence on marketing/advertisements
Promotion of unhealthy foods in supermarkets
TV advertising plays and important role in determining products purchased by parents ‘pester power’
Increased accessibility of multinational low-priced food chains, the increase in street food vendors selling energy dense processed foods in poor areas, and the increased consumption of sugar-sweetened beverages
WHO recommendations:
• tightening up existing (voluntary) codes of practice;
• increasing promotional activity for healthier food products to redress the current imbalance;
• providing guidance to schools and other educational establishments to help encourage healthy eating;
• introducing statutory controls on the promotion of foods to children (for example, banning food advertising to children of a certain age)
Promotion of healthy foods
Access and use of TV
Exposure to advertising
Unhealthy food available and accessible
Regulation
Outcomes
Unhealthy food marketing to children.
Preferences for unhealthy foods increase
Increase overall consumption of unhealthy foods leading to increased rates of obesity and non-communicable diseases
Less exposure to unhealthy food advertisements
Reduction in household purchases of ‘fast food’
Reduced consumption of less healthy foods
+
Increased consumption of healthier foods
=Improved diet leading to reduced rates of obesity and non-communicable diseases
