# Supplementary material for: Policy Action Within Urban African Food Systems to Promote Healthy Food Consumption: A Realist Synthesis in Ghana and Kenya
Source: Int J Health Policy Manag. 2021 Feb 9;10(12):828–44. doi: 10.34172/ijhpm.2020.255 (PMC9309963; doi:10.34172/ijhpm.2020.255)
Supplement: Supplementary file 6 — Pathway From Food Promotion/Regulation in Schools to Reduced Consumption of Unhealthy Foods. [file ijhpm-10-828-s006.pdf]

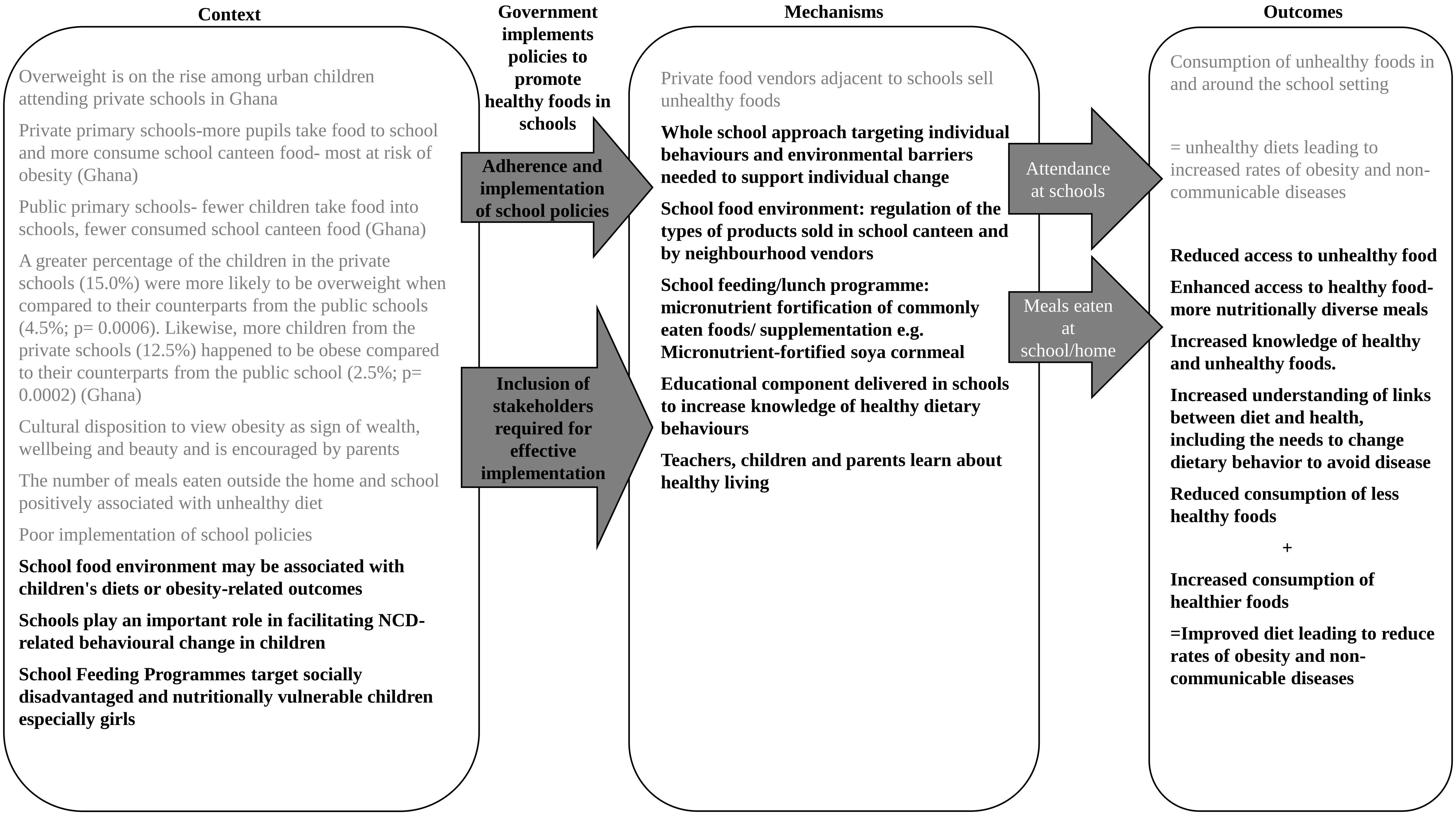
Context
Government implements policies to promote healthy foods in schools
Mechanisms
Outcomes
Overweight is on the rise among urban children attending private schools in Ghana
Private primary schools-more pupils take food to school and more consume school canteen food- most at risk of obesity (Ghana)
Public primary schools- fewer children take food into schools, fewer consumed school canteen food (Ghana)
A greater percentage of the children in the private schools (15.0%) were more likely to be overweight when compared to their counterparts from the public schools (4.5%; p= 0.0006). Likewise, more children from the private schools (12.5%) happened to be obese compared to their counterparts from the public school (2.5%; p= 0.0002) (Ghana)
Cultural disposition to view obesity as sign of wealth, wellbeing and beauty and is encouraged by parents
The number of meals eaten outside the home and school positively associated with unhealthy diet
Poor implementation of school policies
School food environment may be associated with children's diets or obesity-related outcomes
Schools play an important role in facilitating NCD-related behavioural change in children
School Feeding Programmes target socially disadvantaged and nutritionally vulnerable children especially girls
Adherence and implementation of school policies
Inclusion of stakeholders required for effective implementation
Private food vendors adjacent to schools sell unhealthy foods
Whole school approach targeting individual behaviours and environmental barriers needed to support individual change
School food environment: regulation of the types of products sold in school canteen and by neighbourhood vendors
School feeding/lunch programme: micronutrient fortification of commonly eaten foods/ supplementation e.g. Micronutrient-fortified soya cornmeal
Educational component delivered in schools to increase knowledge of healthy dietary behaviours
Teachers, children and parents learn about healthy living
Attendance at schools
Meals eaten at school/home
Consumption of unhealthy foods in and around the school setting
= unhealthy diets leading to increased rates of obesity and non-communicable diseases
Reduced access to unhealthy food
Enhanced access to healthy food- more nutritionally diverse meals
Increased knowledge of healthy and unhealthy foods.
Increased understanding of links between diet and health, including the needs to change dietary behavior to avoid disease
Reduced consumption of less healthy foods
+
Increased consumption of healthier foods
=Improved diet leading to reduce rates of obesity and non-communicable diseases
